# Effect of dynamic contact angle variation on spontaneous imbibition in porous materials

**DOI:** 10.1007/s11242-022-01754-y

**Published:** 2022-03-03

**Authors:** Michele Bianchi Janetti, Hans Janssen

**Affiliations:** 1 Unit for Energy Efficient Building, Institute for Structural Engineering and Material Sciences, University of Innsbruck (AT), Technikerstrasse 13, 6020 Innsbruck, Austria; 2 Department of Civil Engineering, Building Physics and Sustainable Design Section, KU Leuven (BE), Kasteelpark Arenberg 40, bus 2447, 3001 Leuven, Belgium

**Keywords:** Spontaneous imbibition, Porous materials, Pore-scale modelling, Dynamic contact angle, Dynamic effects

## Abstract

We investigate the influence of contact angle variations on spontaneous imbibition of moisture in porous materials. While the contact angle is typically assumed constant when modelling the moisture transfer in porous media, experimental findings put this assumption into question. It has been shown that during imbibition the contact angle notably rises with increasing meniscus velocity. This phenomenon resultantly affects the moisture retention curve, the relation linking the local capillary pressure to the local moisture saturation, which in turn impacts the imbibition rate and moisture distribution. This study investigates these dynamic effects via a pore network technique as well as a continuum approach. It is shown that the impacts of pore-scale contact angle variations on the imbibition process can be reproduced at the continuum scale through a modified moisture retention curve including a dynamic term. Complementarily a closed-form equation expressing the dynamic capillary pressure in terms of local saturation and saturation rate is derived. The continuum approach is then finally employed to predict measured moisture saturation profiles for imbibition in Berea sandstone and diatomite found in literature, and a fair agreement between simulated and measured outcomes is observed.

## Introduction

Moisture transport and storage in porous materials play an important role in many fields of science and engineering such as concrete technology, soil science, geology, hydrology and building physics. The availability of suitable numerical models for a reliable assessment of the moisture distribution in porous materials is therefore crucial in all these disciplines. To this aim, a continuum approach is often employed, which implies the existence of a representative elementary volume where average (local) moisture states and material properties are defined (Künzel and Kiessl 1996; Häupl et al. 1997; Janssen et al. 2007; Bianchi Janetti et al. 2018). Accordingly, the material’s capability for storing and transporting moisture due to capillary forces is described by empirical material properties, i.e., the moisture retention and moisture permeability curves. The former establishes a relationship between the local capillary pressure and the local moisture saturation, the latter expresses the local moisture permeability as a function of the local moisture saturation. Often these material functions are assumed to be independent of the (de)saturation rate (speed of local saturation changes over time), by describing the moisture transfer via the well-known diffusion equation. Experimental findings reveal that this assumption may not always be valid though, having shown that the (de)saturation rate may significantly influence the moisture retention curve (Hassanizadeh and Gray 1993; Carroll et al. 2010; Janssen et al. 2016; Bianchi Janetti and Janssen 2020; Hassanizadeh et al. 2002; Joekar-Niasar and Hassanizadeh 2011). The occurrence of this phenomenon, which is still not fully explained, is addressed in the literature as “dynamic effects” on the moisture retention curve. Such dynamic effects can be taken into account by defining a local dynamic capillary pressure, being a function of both local saturation and saturation rate. Generally, this empirical relation is used (Hassanizadeh and Gray 1993; Carroll et al. 2010; Janssen et al. 2016; Bianchi Janetti and Janssen 2020; Hassanizadeh et al. 2002; Joekar-Niasar and Hassanizadeh 2011):

1 $$P_{c,d} \left( {S,{\dot{S}}} \right) = P_{c,e} \left( S \right) - \tau {\dot{S}}$$

here $P_{c,e}$ [Pa] denotes the local static (equilibrium) capillary pressure, $P_{c,d}$ [Pa] the local dynamic capillary pressure, depending on both local saturation $S$ [-] and saturation rate $\dot{S} = \partial S/\partial t$ [1/s], and $\tau$ [Pa s] a parameter usually referred to as the dynamic storage coefficient. Equation (1) reflects the experimental evidence that dynamic effects vanish for sufficiently slow processes ($\dot{S} \to 0$), reducing the moisture retention curve to a function of local saturation only: $P_{c,d} \left( {S,0} \right) = P_{c,e} \left( S \right)$. The equation presumes that the deviations between dynamic and static local capillary pressure are proportional to the saturation rate via the dynamic storage coefficient, which is empirically determined by inverse fitting of experimental data (Carroll et al. 2010; Janssen et al. 2016; Bianchi Janetti and Janssen 2020) or with pore-scale simulations (Hassanizadeh et al. 2002; Joekar-Niasar and Hassanizadeh 2011). Various studies suggest this coefficient being related to the fluid properties (viscosity, density, interfacial tension) (Hassanizadeh and Gray 1993; Joekar-Niasar and Hassanizadeh 2011), the material properties (porosity and pore size distribution) (Hassanizadeh and Gray 1993) as well as the pore surface wettability (contact angle of fluid and solid) (Hassanizadeh and Gray 1993; Carroll et al. 2010).

Dynamic effects have been investigated in Carroll et al. (2010) via drainage experiments, showing that contact angle variations due to change of the drainage velocity highly impact the moisture retention curve. On the other hand, dynamic effects reveal themselves during spontaneous imbibition as the deviation from the expected square-root-of-time behaviour of the imbibition process (Guen and Kovscek 2006; Zahasky and Benson 2019; Zhao et al. 2018). While this phenomenon has been empirically observed, a sound mathematical model is to the authors’ best knowledge still missing.

The present paper contributes to overcoming this lack of knowledge, with focus on the spontaneous imbibition process. The main innovation refers to the upscaling approach employed to describe the dynamic effect and the link established between dynamic contact angle at the capillary level and such macroscopic dynamic effect. While previous studies (Hassanizadeh and Gray 1993; Carroll et al. 2010; Janssen et al. 2016; Bianchi Janetti and Janssen 2020; Hassanizadeh et al. 2002; Joekar-Niasar and Hassanizadeh 2011) express the local dynamic capillary pressure $P_{c,d} \left( {S,\dot{S}} \right)$ in a purely empirical manner, we suggest here an alternative closed-form equation expressing the dynamic local capillary pressure as a function of local saturation and saturation rate. This equation is obtained by upscaling the contact-angle-meniscus-velocity relationship defined at the capillary level. The proposed model provides better insight into how fluid viscosity, surface tension, surface wettability and pore structure impact on the dynamic effects. Subsequently, the proposed model is used to describe spontaneous imbibition in an artificial material sample at the continuum scale. The same imbibition process is simulated with a pore network model in which dynamic contact angles are implemented. The saturation profiles obtained from the pore network simulations are hence used to validate the continuum model. Further testing is carried out by comparing the continuum simulations with measured saturation profiles during imbibition in diatomite and Berea sandstone found in the literature. It appears that pore-scale dynamic contact angle variation may indeed explain dynamic effects observed at the continuum scale.

## Capillary pressure as a function of the dynamic contact angle

**Fig. 1:**
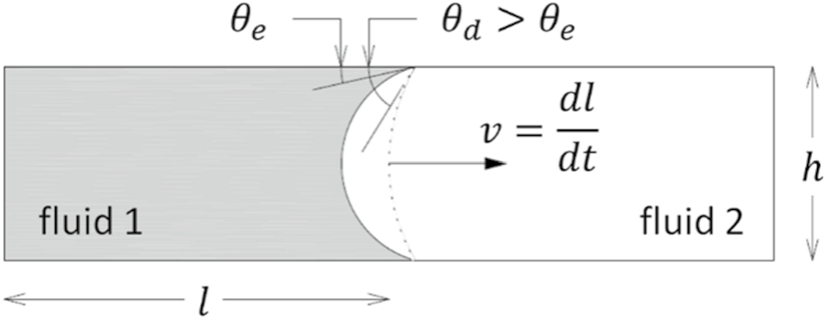
Slit formed by two parallel plates containing a wetting fluid 1 (liquid water) and a non-wetting fluid 2 (air). The dotted line denotes the fluid–fluid interface during spontaneous imbibition and the solid line denotes the meniscus at equilibrium

In this section, a relation linking the pore-scale capillary pressure to the dynamic contact angle is introduced, for which the partially saturated slit enclosed by parallel plates shown in Fig. 1 is considered as exemplary geometry.

The capillary pressure ${p}_{c}$ [Pa] is defined as the pressure drop across the meniscus:

2 $$p_{c} = p_{w} - p_{{{\text{nw}}}}$$

here ${p}_{w}$ [Pa] and ${\mathrm{p}}_{\mathrm{nw}}$ [Pa] denote the pressure in the wetting and non-wetting phase (liquid water and air), respectively. The capillary pressure can be expressed as a function of the slit width and contact angle via the well-known Young–Laplace equation:

3 $$p_{c} = - \frac{2\sigma \cos \left( \theta \right)}{h}$$

here $\sigma$ [N/m] denotes the surface tension of the wetting fluid, $h$ [m] the slit width and $\theta$ [°] the contact angle between the wetting fluid and the solid surface. This contact angle may change depending on the motion of the meniscus. Experimental investigations of imbibition processes have shown that the contact angle increases with increasing meniscus velocity $v$ [m/s] (Carroll et al. 2010; Heshmati and Piri 2014; Li et al. 2013; Sobolev et al. 2000). At equilibrium (for a static meniscus) the contact angle assumes it’s minimum value $\theta_{d} \left( {v = 0} \right) = \theta_{e}$, with $\theta_{d} \left( v \right)$ [°] the contact angle for the advancing meniscus (dynamic contact angle).

In the literature (Carroll et al. 2010; Heshmati and Piri 2014; Li et al. 2013), there are several expressions for dynamic contact angle $\theta_{d} \left( v \right)$, empirical or derived from hydrodynamics and molecular kinetics. These expressions all confirm that dynamic contact angle is governed by the capillary number ${\text{Ca}} = \eta v/{\sigma}$, where $\eta$ [Pa s] denotes the dynamic viscosity and $\sigma$ [N/m] the surface tension of the wetting fluid. In this work the following Eq. (4) taken from Li et al. (2013) is used, although the proposed approach can be easily adapted to other $\theta_{d} \left( v \right)$ relations:

4 $$\cos \left( {\theta_{d} } \right) = \cos \left( {\theta_{e} } \right) - A\left[ {1 + \cos \left( {\theta_{e} } \right)} \right]\left( {\frac{\eta v}{\sigma }} \right)^{B}$$

here $\theta_{e}$, A, B are empirical parameters which must be determined by fitting experimental data.

It is worth to note that Eq. (4) can only be applied for $0 \le v < {\tilde{v}}$, with $\theta_{d} \left( {\tilde{v}} \right) = \pi /2{ }$. Out of this range ($v \ge \tilde{v}$) Eq. (4) delivers values of the dynamic contact angle $\theta_{d} \ge \pi /2$ which obviously do not have any physical meaning. To complete the model, the contact angle is hence assumed to remain constant, at a value slightly smaller than $\pi /2$, for any $v \ge \tilde{v}$. Do keep in mind though that this case occurs in practice just in the very initial stages of imbibition. Later on, the process slows down and the meniscus velocities drop to the range in which Eq. (4) can be safely applied.

**Fig. 2:**
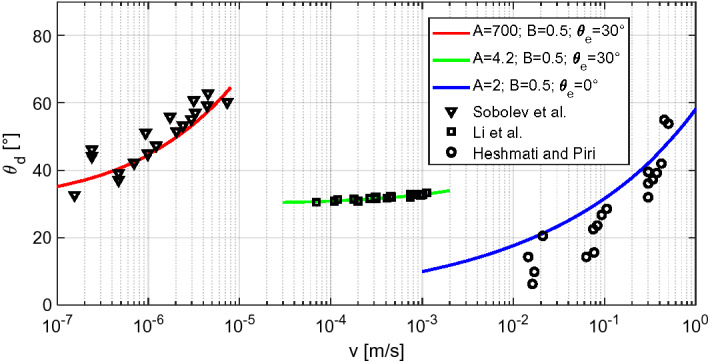
Dynamic contact angle measured by Heshmati et al. (Heshmati and Piri 2014), Li et al. (Li et al. 2013) and Sobolev et al. (Sobolev et al. 2000). The measured values are fitted with Eq. (4) (solid lines)

Some results concerning imbibition experiments of liquid water in capillaries are reported in Fig. 2. In (Heshmati and Piri 2014), measurements of imbibition in glass capillaries with internal diameters varying from 0.75 to 1.3 mm are documented. For meniscus velocities up to 0.6 m/s, fair agreement is found with $\theta_{e} = 0^\circ$, *A* = 2 and *B* = 0.5. More data are brought in Li et al. (2013), with glass capillaries with diameters varying from 100 to 250 µm and meniscus velocities up to 0.0012 m/s. In that study, optimal agreement is found with $\theta_{e} = 30^\circ$, *A* = 4.2 and *B* = 0.51. Imbibition in quartz capillaries of diameters varying from 90 to 544 nm is presented in Sobolev et al. (2000). Contrary to the works cited above (Heshmati and Piri 2014; Li et al. 2013), in Sobolev et al. (2000) contact angles are velocity-dependent only for *v* < 5 µm/s and remain nearly constant above that value, with Eq. (4) with $\theta_{e} = 30^\circ$, *A* = 700 and *B* = 0.5 yielding a fair fit. The major deviation of the *A* coefficient from the earlier values can be explained by considering the drastically smaller radii of the considered capillaries and the resultantly far lower meniscus velocities analyzed in the latter study.

In general, it must be noted that any $\theta_{d} \left( v \right)$ relationship presents limited validity, depending on the meniscus velocity and capillary size, and has to be carefully chosen according to the range of application of these parameters. Additional consideration shall be made with respect to the equilibrium contact angle, characterizing the wettability of the solid surface when the meniscus velocity approaches zero. $\theta_{e} = 0^\circ$ is often assumed for moisture transfer in porous media with silica as dominant constituent (Carroll et al. 2010; Anderson 1986). This assumption may, however, be unsuitable for materials as calcite, dolomite, coal or talc (Carroll et al. 2010; Anderson 1986), for surfaces coated with natural organic material (Carroll et al. 2010; Ryder and Demond 2008), or for surfaces that have been exposed to surfactants or NAPLs (Carroll et al. 2010; Ryder and Demond 2008).

From Eqs. (3) and (4) one obtains the dynamic capillary pressure ${p}_{c,d}$ as a function of the slit width and meniscus velocity as follows:

5 $$p_{c,d} \left( {h,v} \right) = p_{c,e} \left( h \right)\left[ {1 - A\frac{{1 + \cos \left( {\theta_{e} } \right)}}{{\cos \left( {\theta_{e} } \right)}}\left( {\frac{\eta v}{\sigma }} \right)^{B} } \right]$$

with $p_{c,d} \left( {h,0} \right) = p_{c,e} \left( h \right)$ defining the static capillary pressure.

## Pore network approach

**Fig. 3:**
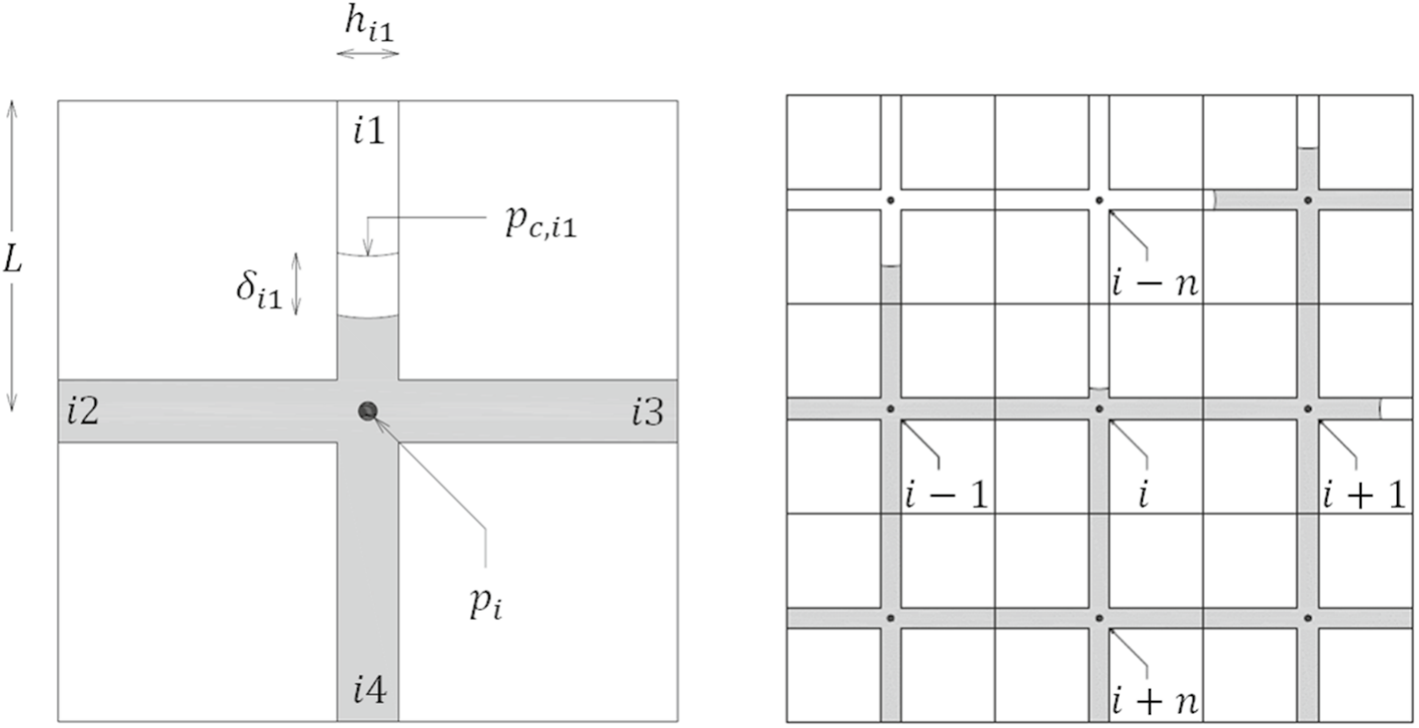
i-th element located in a network composed by $n$ columns. Each element presents 4 throats of thickness $h_{ij}$. Two neighbouring throats in adjacent network elements are forced to have the same thickness, i.e., for each throat $ij$ holds: $h_{i1} = h_{{\left( {i - n} \right)4}}$; $h_{i2} = h_{{\left( {i - 1} \right)3}}$; $h_{i3} = h_{{\left( {i + 1} \right)2}}$; $h_{i4} = h_{{\left( {i + n} \right)1}}$

In order to investigate the effect of the contact angle variation on the imbibition process, a pore network model, similar to the one described in Gruener et al. (2012), is implemented. Such model represents a porous material, in which capillary driven moisture storage and flow occurs. The network nodes are located on a two-dimensional cartesian grid, see Fig. 3, and are connected by throats of equal length which are in fact slits enclosed between infinitely extended parallel plates.

The entire pore volume in the grid is assigned to the throats, while the nodes themselves are assumed to be point-like and without volume. Piston flow is imposed in each (partially) filled throat. Pressure gradients in the non-wetting fluid (air) as well as phase change processes (evaporation and condensation) at the water–air interface are hence disregarded. Assuming laminar flow, the momentum equation reduces to the plane Hagen-Poiseuille’s law, which describes the mass flow $j_{ij}$ [kg/s] trough the throat $ij$ as follows:

6 $$j_{ij} = - \frac{{\rho h_{ij}^{2} }}{12 \eta }\frac{{\Delta p_{ij} }}{{l_{ij} }}A_{ij}$$

here $l_{ij}$ [m] denotes the length of the liquid column in the throat $ij$, $\rho$ [kg/m^3^] the density of liquid water, $h_{ij}$ [m] the thickness of the throat, $A_{ij}$ [m^2^] the cross section area of the throat and $\Delta p_{ij}$ [Pa] the pressure drop across the liquid column. For completely filled throats $l_{ij} = 2L$, $2L$ [m] being the distance between two neighbouring nodes. The spatial pressure distribution in the network at the time $t$ is hence determined by solving a system of equations derived from the liquid water conservation at each water-filled node $i$:

7 $$\sum\nolimits_{j = 1}^{4} {j_{ij} = 0}$$

$j_{ij}$ being the moisture flows [kg/s] from and to the node $i$. To proceed from the current time step $t$ [s] to the next time step $t + \Delta t$ [s], the menisci positions are updated by calculating the advancing distance $\delta_{ij}$ [m] of each meniscus as follows:

8 $$\delta_{ij} = - \frac{{h_{ij}^{2} }}{12 \eta }\frac{{p_{c,ij} - p_{i} }}{{l_{ij} }}\Delta t.$$

here ${p}_{i}$ [Pa] denotes the pressure at the node $i$ and ${p}_{c,ij}$ [Pa] the capillary pressure. All these quantities are determined at the time $t$. The new length is then explicitly calculated as follows:

9 $$l_{ij} \left( {t + \Delta t} \right) = l_{ij} \left( t \right) + \delta_{ij} \left( t \right).$$

Note that, according to Eq. (5), the capillary pressure ${p}_{c,ij}$ depends on the meniscus velocity, which is given by:

10 $$v_{ij} = \frac{{\delta_{ij} }}{\Delta t}$$

The invasion algorithm is completed by a few rules which control the meniscus propagation: (1) when the meniscus reaches a still inactive node, this is activated by setting the liquid length in the connected throats to a small value $l_{ij0}$ before evaluating the new pressure distribution (in the case study shown below, $l_{ij0} = L/40$ gives fair results in terms of mass conservation); (2) if the pressure difference $p_{i} - p_{c,ij}$ in a throat is negative, then the meniscus is arrested until such pressure difference becomes positive again; (3) when two menisci meet, they merge (i.e., air entrapment is disregarded).

## Continuum approach

The continuum approach used for modelling moisture transfer in unsaturated porous materials is based on the definition of a representative elementary volume in which average (local) moisture states and material properties are defined. Compared to the pore network model, this approach requires a lower computational cost, but it on the other hand does not allow insight into the wetting dynamics of single pores. By neglecting gravity effects as well as the pressure gradients in the non-wetting phase (air), and by defining the gradient of local capillary pressure equal to the pressure gradient in the wetting phase (liquid water), the mass balance equation for the one-dimensional case becomes (Guen and Kovscek 2006):

11 $$\psi \rho \frac{\partial S}{{\partial t}} = \frac{\partial }{\partial x}\left( {K\frac{{\partial P_{c} }}{\partial x}} \right)$$

here $\psi$[−] denotes the porosity, $\rho$[kg/m^3^] the density of liquid water, $S$ [−] the local moisture saturation, $K$ [kg/(m s Pa)] the permeability and ${P}_{c}$ [Pa] the local capillary pressure. To complete the model, a relation linking the local capillary pressure to the local saturation (and saturation rate) is required. Note that, in absence of dynamic effects, the local capillary pressure is a function of local saturation only and Eq. (11) turns into the well-known diffusion equation. Here, the dependency of the local capillary pressure on the local saturation rate is taken into account by replacing in Eq. (5) the pore-scale capillary pressure ${p}_{c}$ and meniscus velocity $v$ with their macroscopic counterparts ${P}_{c}$ and $U$. Accordingly, we obtain:

12 $$P_{c,d} (S,U) = P_{c,e} \left( S \right) \left[ 1 - A \frac{{1 + \cos \left( {\theta_{e} } \right)}}{{\cos \left( {\theta_{e} } \right)}}\left( {\frac{\eta U}{\sigma }} \right)^{B} \right].$$

with $P_{c,d} \left( {S,0} \right) = P_{c,e} \left( S \right)$ [Pa] representing the local equilibrium capillary pressure, i.e. the capillary pressure in absence of dynamic effects, and $U$ [m/s] the average local meniscus velocity, which must satisfy the following equation:

13 $$A_{{{\text{nw}}}} U = V_{p} \dot{S}$$

with $A_{{{\text{nw}}}}$ [m^2^] the surface of active menisci (liquid–gas interface) and $V_{p}$ the total pore volume [m^3^] in a representative material element. The product $A_{{{\text{nw}}}} U$ represents the increase of the volume occupied by the liquid when the liquid–gas interface is advancing with a uniform velocity $U$. This quantity has to be equal to the temporal change of the liquid volume ($V_{p} \dot{S}$) in the same representative element of volume. We can hence express the average (local) velocity as follows:

14 $$U = \frac{{V_{p} \dot{S}}}{{A_{{{\text{nw}}}} }} = \frac{{\dot{S}}}{\xi }$$

where $\xi$ [1/m] represents the ratio of the liquid–gas interface to the total pore volume in a representative material element:

15 $$\xi \left( S \right) = \frac{{A_{{{\text{nw}}}} \left( S \right)}}{{V_{p} }}.$$

Note that $A_{{{\text{nw}}}} \left( S \right)$ and $\xi \left( S \right)$ are functions of the local saturation $S$, being equal to zero for $S = 0$ and for $S = 1$ and positive elsewhere. By taking into account Eq. (14), we write Eq. (12) as follows:

16 $$P_{c,d} \left( {S,\dot{S}} \right) = P_{c,e} \left( S \right) - AP_{c,e} \left( S \right)\frac{{1 + \cos \left( {\theta_{e} } \right)}}{{\cos \left( {\theta_{e} } \right)}}\left[ {\frac{{\eta \dot{S}}}{\sigma \xi \left( S \right)}} \right]^{B}$$

which in compact form reads:

17 $$P_{c,d} \left( {S,\dot{S}} \right) = P_{c,e} \left( S \right) - \tau^{*} \left( S \right) \dot{S}^{B}$$

$\tau^{*} \left( S \right)$ [Pa s^B^] being defined as follows:

18 $$\tau^{*} \left( S \right) = AP_{c,e} \left( S \right)\frac{{1 + \cos \left( {\theta_{e} } \right)}}{{\cos \left( {\theta_{e} } \right)}}\left[ {\frac{\eta }{\sigma \xi \left( S \right)}} \right]^{B}$$

Note that for $B = 1$ Eq. (17) turns into the well-known linear correlation expressed by Eq. (1) and $\tau^{*} = \tau$. However, while Eq. (1) is a purely empirical correlation, Eq. (17) has been derived through a mathematical procedure starting from pore-scale relationships. In this way, the dependency of the dynamic storage coefficient on the fluid properties (viscosity and surface tension), equilibrium contact angle, equilibrium capillary pressure and ratio of the liquid–gas interface to the total pore volume can be explicitly formulated. Although proper validation of Eq. (18) is currently precluded by the lack of suitable experimental data, the model confirms some general trends already observed in the literature. Equation (18) suggests that for fine-pore materials presenting high $P_{c,e} \left( S \right)$ and low permeability, the dynamic effects would be larger. This is in agreement with the statements in Hassanizadeh et al. (2002) and outcomes discussed below in Sect. 6. The fact that the dynamic storage coefficient rises with the increasing viscosity of the wetting fluid was also already observed in Hassanizadeh et al. (2002).

## Exemplary case study on artificial material

### Model definition and material properties

**Fig. 4:**
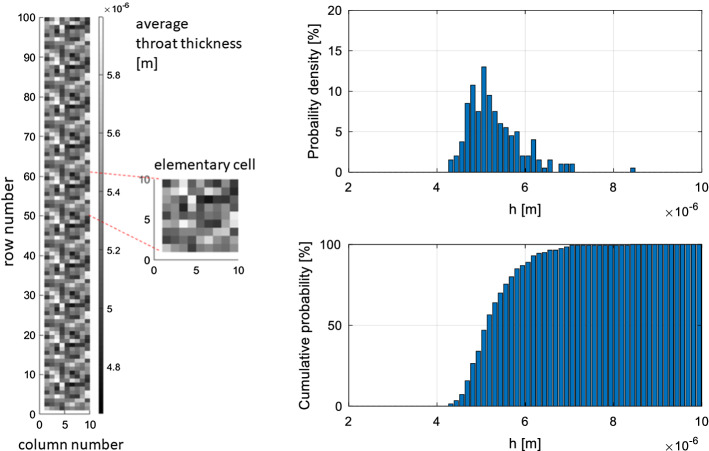
Throat size distribution. Left: throat thickness distribution in the network of 10 × 100 elements, the colour scale refers to the average throat thickness of each network element; right: probability density function and cumulative probability

The pore network method and the continuum approach are verified by simulating spontaneous imbibition in an artificial material sample. This sample comprises 10 equal elementary cells placed on top of each other, each cell including 10 × 10 square network elements of side $2L = 20$ µm. Each network element is composed by four throats connected to a central node, as shown above in Fig. 3. The throat thicknesses are randomly assigned from a generalized extreme value distribution. The porosity of the artificial material is: $\psi = 0.52 \left[ - \right].$ In Fig. 4, the throat thickness distribution in the sample as well as the probability density and cumulative probability of throat thickness are shown. The network elements placed at the sample bottom are in contact with the water reservoir, whereas periodic boundary conditions are imposed at both long sides of the sample. Imbibition occurs according to the invasion algorithm described above in Sect. 3.

The material functions $P_{c,e} \left( S \right)$, $K\left( S \right)$ and $\xi \left( S \right)$ are determined at equilibrium ($\dot{S} = 0$), i.e., by applying a quasi-static invasion algorithm on a single elementary cell representative of the entire network. This quasi-static invasion algorithm is briefly described as follows: at each given value of saturation $S$ all throats of thickness $h_{ij} < h\left( S \right)$ are assumed to be completely filled of water, while the remaining ones are empty. The one-to-one relationship linking the local capillary pressure to the local saturation, i.e., $P_{c,e} \left( {h\left( S \right)} \right)$, is hence directly defined taking into account Eq. (3). We might then determine the elementary cell’s permeability $K\left( S \right)$ as the ratio of the total stationary flow through the cell to the pressure drop over the cell itself. Since just the filled throats contribute to the flow, the permeability increases by increasing saturation. Finally, the interface area $A_{{{\text{nw}}}} \left( S \right)$ between liquid water and air is determined as the sum of all interfaces in the elementary cell, the function $\xi \left( S \right)$ being defined by Eq. (15).

**Fig. 5:**
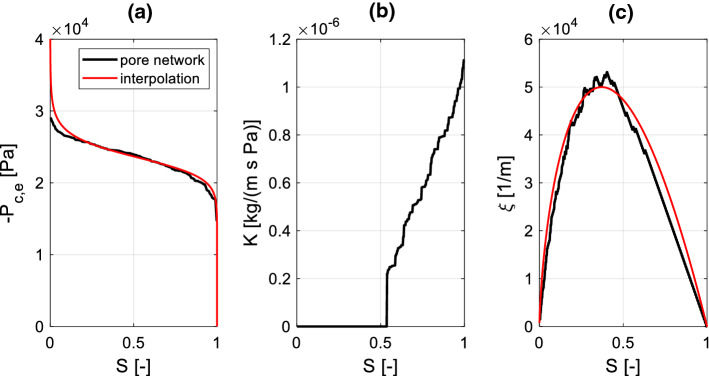
Material functions for continuum simulation: **a** equilibrium moisture retention curve determined for $\theta_{e} = 30^\circ$, **b** permeability curve and **c** ratio of the liquid–gas interface to the total pore volume

**Fig. 6:**
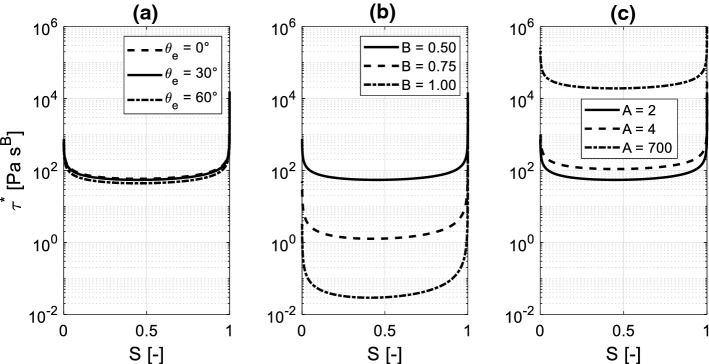
Dynamic storage coefficient determined with Eq. (18): **a** impact of the equilibrium contact angle (*A* = 2 and *B* = 0.5); **b** impact of the parameter *B* ($\theta_{e} = 30^\circ$ and *A* = 2) and **c** impact of the parameter *A* ($\theta_{e} = 30^\circ$ and *B* = 0.5)

The material functions, shown in Fig. 5, are used as inputs of the continuum approach for simulation of spontaneous imbibition. Since the continuum approach requires smooth functions for $P_{c,e} \left( S \right)$ and $\xi \left( S \right)$ the raw network data obtained from the pore network have been fitted with Eq. (19) (Genuchten 1980) and Eq. (20), respectively:

19 $$P_{c,e} \left( S \right) = a_{1} \left( {\sqrt[{a_{2} - 1}]{S} - 1} \right)^{{a_{2} }}$$

20 $$\xi \left( S \right) = b_{1} \left[ {1 - \left( {2S^{{b_{2} }} - 1} \right)^{2} } \right]$$

where $a_{i}$, $b_{j}$ are empirical parameters. The coefficient $\tau^{*} \left( S \right)$ is hence derived from $P_{c,e} \left( S \right)$ and $\xi \left( S \right)$ according to Eq. (18). The effects of the equilibrium contact angle $\theta_{e}$ and of the parameters A and B on the dynamic storage coefficient $\tau^{*} \left( S \right)$ are shown in Fig. 6. It appears that $\theta_{e}$ has just a minor impact, while parameters A and B might highly influence the curve $\tau^{*} \left( S \right)$. Note that, all experimental studies reviewed in Sect. 2 indicate *B* = 0.5 for liquid water, while the parameter A might vary in a range between 2 and 700. The parameter A controls therefore the relationship linking the contact angle to the meniscus velocity given by Eq. (4) and accordingly the magnitude of $\tau^{*} \left( S \right)$ at the macroscopic scale.

### Results of moisture imbibition simulations

**Fig. 7:**
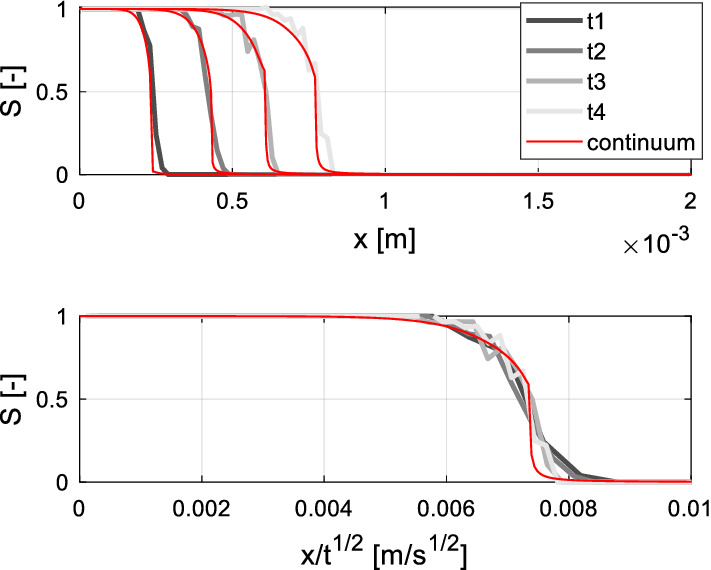
Saturation profiles obtained by the pore network and continuum approach with static contact angle $\theta = 30^\circ$. *t*1 = 1.1; *t*2 = 3.3; *t*3 = 6.5; *t*1 = 10.0 [ms]

In this section, saturation profiles obtained from the continuum model are compared to the pore network results. In a first case, the contact angle is assumed constant over the entire imbibition process, equal to the equilibrium contact angle ($\theta_{e} = 30^\circ$ is assumed). Accordingly, the local capillary pressure becomes a function of local saturation only and Eq. (11) turns into the normal diffusion equation. The saturation profiles along the sample axis, as determined with the pore network and continuum model, are shown at four different times in Fig. 7. In conformity with diffusion theory (Crank 1975; Bianchi Janetti and Wagner 2017), the saturation profiles obtained from the continuum approach collaps into a single curve, when expressed as function of the Boltzmann variable $\lambda = x/t^{1/2}$ (bottom graph of Fig. 7). It can be observed that also the pore network profiles nearly transform into a single curve, hence indicating that dynamic effects are negligible when the contact angle is constant during the entire process. Fair agreement is observed between the outcomes of the pore network and the continuum models.

**Fig. 8:**
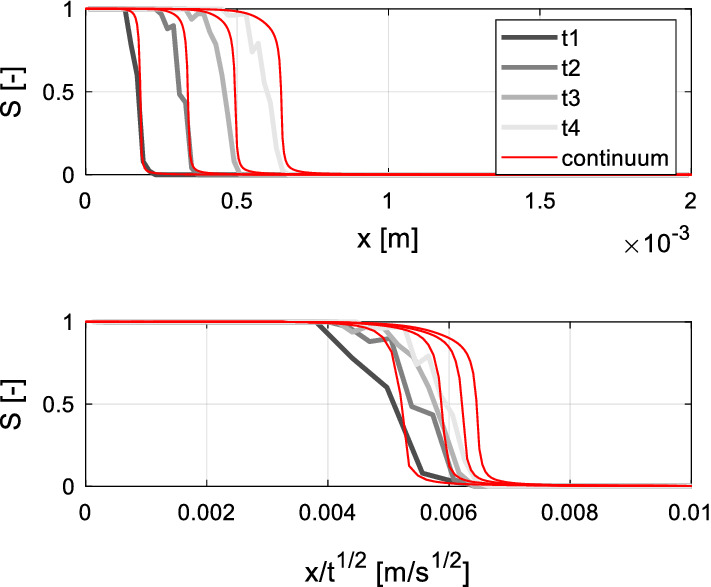
Saturation profiles obtained by the pore network and continuum approach with dynamic contact angle according to Eq. (4) ($\theta_{e} = 30^\circ$; *A* = 2; *B* = 0.5). *t*1 = 1.1; *t*2 = 3.3; *t*3 = 6.5; *t*1 = 10.0 [ms]

A second test is carried out by applying a dynamic (variable) contact angle in the pore network model. In the continuum approach, contact angle variations are taken into account through the dynamic capillary pressure expressed by Eq. (17). The results of both the continuum and pore network model are reported in Fig. 8. Here, a delay of the imbibition process is noted, relative to the earlier case with constant angle: the moisture saturation profiles are shifted to the left side of the graph. This is consistent with the fact that the dynamic contact angle is larger than the static one, thus leading to a smaller capillary pressure and accordingly slower imbibition. When a variable contact angle is used, the saturation can no longer be expressed as a unique function of the Boltzmann variable, implying that the normal diffusion equation cannot adequately capture the imbibition process (Fig. 8, bottom graph). Instead, the continuum model proposed above predicts the delay of the moisture saturation front in fair agreement with the pore network algorithm, confirming the adequateness of the upscaling procedure applied in Sect. 4.

Although the fundamental imbibition behaviour is similar in both models, small deviations between pore network and continuum approach are present and require some comment. These deviations observed in the saturation distribution (especially in Fig. 8) might be attributed to imperfections of the upscaling method applied to determine the macroscopic material functions. In this regard, let us note here that the steady-state algorithm employed to determine $A_{{{\text{nw}}}} \left( S \right)$ computes the entire liquid–gas interface, while for better accuracy only active (advancing) menisci should be included (static menisci do not contribute to the flow). This fact leads to an overestimation of $\xi \left( S \right)$ up to circa 40%. The fact that $P_{c,e} \left( S \right)$ and $\xi \left( S \right)$ are constituted by discrete values, requiring interpolation to be used for continuum simulation, might lead to further deviations between pore network and continuum results. On the other hand, it has been verified that numerical errors play just a minor role in both continuum and pore network simulation. The above mentioned deviations might thus be reduced, for instance, by optimizing the employed invasion algorithms and by improving interpolation of the material functions. Such tasks overcome the scope of this paper and are hence left for future research.

## Application of model on experimental data

The continuum model is subsequently applied to predict experimental results reported in Guen and Kovscek (2006) and (Zahasky and Benson 2019) concerning spontaneous imbibition in diatomite (porosity $\psi = 0.65 \left[ - \right]$) and Berea sandstone ($\psi = 0.195 \left[ - \right]$), respectively. In both studies, liquid water is employed as wetting phase, while, respectively, air and carbon dioxide form the non-wetting phase. The pressure conditions imposed in Zahasky and Benson (2019) lead to a maximum water saturation (at the inlet location) of approximately 60%, while in Guen and Kovscek (2006) 100% water saturation is obtained. In both studies, the water saturation profiles were determined at different times via X-ray computerized tomography.

**Fig. 9:**
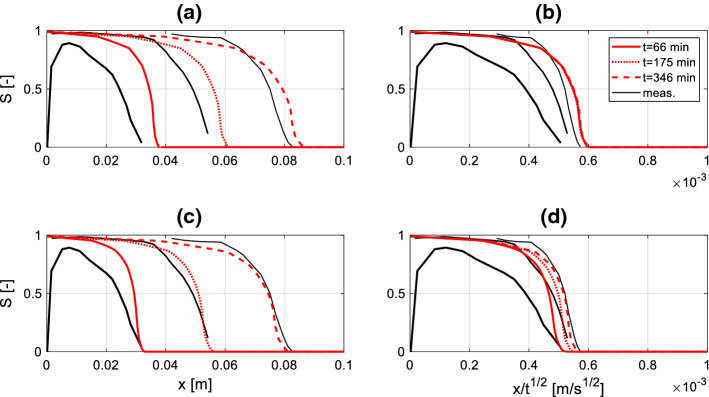
Simulated and measured (Guen and Kovscek 2006) moisture saturation profiles during spontaneous imbibition in diatomite. Graphs **a** and **c** show the moisture saturation as a function of the position along the sample axis, while graphs **b** and **d** show the moisture saturation as a function of the Boltzmann variable. In **a**, **b** the simulated profiles are obtained assuming a static contact angle, in **c**, **d** dynamic contact angle is assumed

**Fig. 10:**
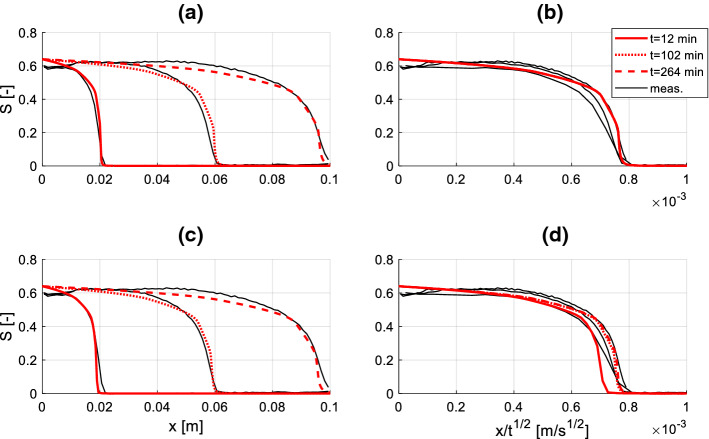
Simulated and measured (Zahasky and Benson 2019) saturation profiles during spontaneous imbibition in Berea sandstone. Graphs **a** and **c** show the moisture saturation as a function of the position along the sample axis, while graphs **b** and **d** show the moisture saturation as a function of the Boltzmann variable. In **a**, **b** the simulated profiles are obtained assuming a static contact angle, in **c**, **d** dynamic contact angle is assumed

**Fig. 11:**
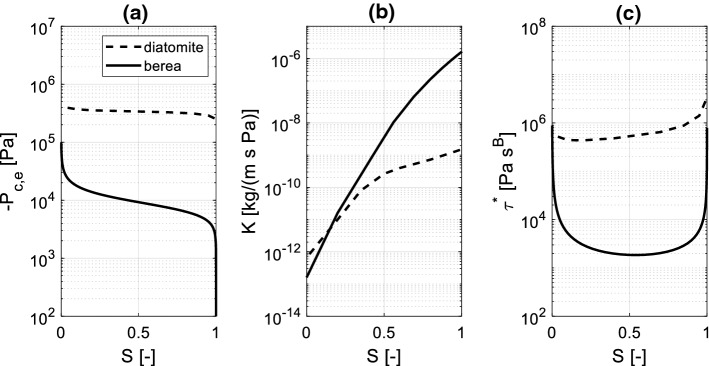
Material functions of Berea sandstone and diatomite; **a** Equilibrium moisture retention curve; **b** permeability curve; **c** dynamic storage coefficient (*B* = 0.5)

The measured and simulated profiles in Berea sandstone and diatomite are reported in Figs. 9 and 10, respectively, while the corresponding material functions used for the simulation are reported in Fig. 11. Among these, the moisture retention curve and permeability curve have been found in the literature (Akin et al. 2000; Schembre-Mc Cabe and Kovscek 2006; Islahuddin and Janssen 2019) while the dynamic storage coefficient is calculated according to Eq. (18) assuming $\theta_{e} = 30^\circ$, *A* = 700 and *B* = 0.5, in agreement with the outcomes of Sobolev et al. (2000) reported in Fig. 2. This choice is motivated considering the low meniscus velocity expected in the pores, which can be roughly estimated by dividing the distance between two moisture fronts by the corresponding time gap. For instance, considering the profiles at 12 min and 102 min in Fig. 10a, c, one deduces average meniscus velocities of approximately 7.4 [µm/s]. At larger times, even lower values of meniscus velocity are estimated.

Since the curve $\xi \left( S \right)$ necessary for calculation of the dynamic storage coefficient is unknown for the considered materials, the relation described by Eq. (20), in which the parameters $b_{1}$ and $b_{2}$ are inversely determined by fitting the measured moisture saturation profiles, is applied as a first approximation. A fair agreement with the measured data is observed for diatomite with $b_{1} = 2.2 \times 10^{4}$ [1/m] and $b_{2} = 0.3$, for Berea sandstone with $b_{1} = 5.5{ } \times 10^{5}$ [1/m] and $b_{2} = 0.7$.

From the graphs (b), (d) on the right sides of Figs. 9 and 10 it appears that the measured profiles do not collapse in a single curve when Boltzmann transformation is applied. This fact suggests that dynamic effects must be taken into account when investigating the imbibition process. To reveal the influence of dynamic effects, two simulations are performed for both materials: in the first variant (graphs (a) and (b)) dynamic effects are neglected (i.e. $P_{c} = P_{c,e} \left( S \right)$ in Eq. (11)), while in the second one (graphs (c) and (d)) dynamic capillary pressure is applied, according to Eq. (17). For both materials, a better agreement with the measured profiles is obtained with the second variant, although the improvement is more evident in case of diatomite. This is reasonably due to the fact that diatomite presents more pronounced dynamic effects on the moisture saturation profiles relative to Berea sandstone, and consequently a much larger dynamic storage coefficient (Fig. 11c). Accordingly, including the dynamic term has a larger impact in case of diatomite relative to Berea sandstone. The observed behaviour appears in agreement with (Hassanizadeh et al. 2002), where it is stated that fine-pore materials with low permeability would present larger dynamic effects in wetting processes.

## Conclusion

We investigate the impact of contact angle variations on spontaneous imbibition in porous media by using a pore network model as well as continuum simulations. The contact angle is assumed to increase with growing velocity of the meniscus, according to an empirical relation for dynamic contact angles reported in the literature. To capture the effect of dynamic contact angle in the continuum approach, a closed-form equation expressing the local capillary pressure as a function of local saturation and saturation rate is obtained by upscaling of the Young–Laplace equation valid at the pore-scale.

The proposed models are tested by simulating the imbibition of water in an artificial material sample. It is shown that, when contact angle variations are neglected, the saturation turns out to be a single function of the Boltzmann variable, as expected in a normal diffusion process. This does not happen when the variation of the contact angle due to decreasing absorption rate (meniscus velocity) is considered. In this case, the saturation profiles at early times are shifted to the left of the graph, i.e., the process results slower than for constant contact angle during the early absorption period. This phenomenon is observed also in measured outcomes found in the literature. The models proposed in this work appear to explain this behaviour.

Accurate understanding and characterization of dynamic effects might be relevant for a number of imbibition processes where high rates of saturation change are expected, such as for water infiltration into dry soil or absorption of wind driven rain in a building facade. In such cases, high dynamic storage coefficient might result into a slower absorption process with crucial impact on the time-dependent moisture distribution. This study aims at taking a step forward towards explaining such dynamic effects on moisture transfer in porous materials. Nevertheless, for the quantitative assessment of this phenomenon on real cases and at the macroscopic scale, a more exhaustive simulation study is required.

## Declarations

### Data availability

The data that support the findings of this study are available from the corresponding author, (Michele Bianchi Janetti) on reasonable request.

### Code availability

The software applications and custom codes that support the findings of this study are available from the corresponding author, (Michele Bianchi Janetti) on reasonable request.
